# Novel Two-Component Systems Implied in Antibiotic Production in *Streptomyces coelicolor*

**DOI:** 10.1371/journal.pone.0019980

**Published:** 2011-05-20

**Authors:** Ana Yepes, Sergio Rico, Antonio Rodríguez-García, Ramón I. Santamaría, Margarita Díaz

**Affiliations:** 1 Instituto de Biología Funcional y Genómica/Departamento de Microbiología y Genética, Consejo Superior de Investigaciones Científicas (CSIC)/Universidad de Salamanca, Edificio Departamental, Campus Miguel de Unamuno, Salamanca, Spain; 2 Instituto de Biotecnología de León, INBIOTEC, Parque Científico de León, León, Spain; University of Wisconsin-Milwaukee, United States of America

## Abstract

The abundance of two-component systems (TCSs) in *Streptomyces coelicolor* A3(2) genome indicates their importance in the physiology of this soil bacteria. Currently, several TCSs have been related to antibiotic regulation, and the purpose in this study was the characterization of five TCSs, selected by sequence homology with the well-known *absA1A2* system, that could also be associated with this important process. Null mutants of the five TCSs were obtained and two mutants (*ΔSCO1744/1745* and *ΔSCO4596/4597/4598*) showed significant differences in both antibiotic production and morphological differentiation, and have been renamed as *abr* (***a***nti***b***iotic ***r***egulator). No detectable changes in antibiotic production were found in the mutants in the systems that include the ORFs *SCO3638/3639*, *SCO3640/3641* and *SCO2165/2166* in any of the culture conditions assayed. The system SCO1744/1745 (AbrA1/A2) was involved in negative regulation of antibiotic production, and acted also as a negative regulator of the morphological differentiation. By contrast, the system SCO4596/4597/4598 (AbrC1/C2/C3), composed of two histidine kinases and one response regulator, had positive effects on both morphological development and antibiotic production. Microarray analyses of the *ΔabrC1/C2/C3* and wild-type transcriptomes revealed downregulation of *actII-ORF4* and *cdaR* genes, the actinorhodin and calcium-dependent antibiotic pathway-specific regulators respectively. These results demonstrated the involvement of these new two-component systems in antibiotic production and morphological differentiation by different approaches. One is a pleiotropic negative regulator: *abrA1/A2*. The other one is a positive regulator composed of three elements, two histidine kinases and one response regulator: *abrC1/C2/C3*.

## Introduction

Antibiotics are highly valuable secondary metabolites that are broadly produced in different species of the genus *Streptomyces*, a filamentous soil bacterium with a complex life cycle. In fact, this genus produces about half of all known microbial antibiotics [1]. The onset of antibiotic production depends on the growth stage of the microorganism and usually takes place contemporaneous with differentiation of the aerial mycelium into spores. Both differentiation and antibiotic production can be triggered by many environmental changes (physical and chemical), such as nutrient deprivation, pH, temperature, etc. These changes must be sensed and integrated in a cell response to promote rapid adaptation to the new growth conditions. The quickest and most efficient bacterial responses to extracellular stimuli occur via histidine- aspartate (His-Asp) phosphorelay cascades. These systems are made up of inner membrane-spanning protein kinases, which sense the external environment, and their respective (cognate) cytoplasmic response regulator partners, which generally exhibit DNA-binding properties. Most of these signal transduction systems only require a single sensor (HK: histidine kinase) and a cognate response regulator (RR) and are thus referred to as two-component systems (TCSs) [2]. Recently, some atypical systems have been described, such as a kinase phosphorylated by GTP instead of ATP [3] and the *p*hosphorylation *i*ndependent *a*ctivation *r*esponse *r*egulators, named PIARR [4]–[6].

*S. coelicolor* A3(2) is the best genetically studied *Streptomyces* strain and has become the model organism for these species. The complete sequence of its 8.7 Mb linear chromosome is available (www.sanger.ac.uk [7]) [8] and contains 84 sensor kinase and 80 response regulator genes, 67 of which lie adjacent on the chromosome and are predicted to form TCSs [9]. The mean HK/RR (TCS) content of *S. coelicolor* (considering the whole 7825 ORFs) is 0.86% as compared with 0.65% for other free-living microorganisms studied or 0.26% for pathogenic bacteria (25% and 70% more in *Streptomyces*, respectively) [10]. This abundance of TCSs could reflect the complexity of the regulatory network of *Streptomyces* that would allow this genus to adapt and survive in multiple and adverse environmental conditions.

*S. coelicolor* A3(2) produces at least four chemically distinct antibiotics: actinorhodin (ACT), undecylprodigiosin (RED), calcium-dependent antibiotic (CDA) and methylenomycin, all of whose biosynthetic genes are located in clusters. The antibiotic production responds to a hierarchy of different levels of decision, distinguishing global or pathway-specific regulators [11]. Pathway-specific regulators are part of the biosynthetic clusters (i.e., *actII-ORF4* for ACT [12]; *redD* for RED [13] and *cdaR* for CDA [14]). Global regulators are located elsewhere and have the ability to regulate operons that belong to different metabolic pathways, and as a consequence mutants in these genes usually show pleiotropic phenotypes. Among the global regulators there are some of which are affecting different process such as differentiation and antibiotic production (i.e. BldA [15], RelA [16], AbsB [17]) and others reported just as global antibiotic regulators (i.e. AbsA1/A2 [18]). TCSs usually act as global regulators that mediate the response from external/internal stimuli to the final target genes.

The function of most of the 67 *S. coelicolor* TCSs is unknown; only a few have an assigned role. Six of them have been reported to modulate the antibiotic production and the best studied, *absA1/A2*, is involved in their global regulation [14], [18]–[21]. The aim of this study was to determine the role of other five TCSs of *S. coelicolor*, whose functions are as yet unknown. Four of them are annotated as homologues to *absA1/A2* in the *Streptomyces* Annotation Server (http://strepdb.streptomyces.org.uk/ [22]). The fifth TCS, although it showed less similarity with *absA1/A2*, is an interesting system because it is composed of two HKs and one RR and may be considered a three-component system. A sequence comparison of this system with the available databases indicated its conservation in almost all the species of *Streptomyces* that are being sequenced by the Broad Institute (http://www.broadinstitute.org/annotation/genome/streptomyces_group/GenomeDescriptions.html).

In this study, the knockouts of the TCSs encoding genes (sensor and regulator at the same time) were generated and the changes in antibiotic production and morphological differentiation were monitored in several medium cultures. Two of the five TCSs selected (*ΔSCO1744/1745* and *ΔSCO4596/4597/4598*) showed significant differences in both antibiotic production and morphological differentiation, and have been renamed as *abr* (***a***nti***b***iotic ***r***egulator). No changes in antibiotic production were detected in the deletion mutants of the other three systems encoded by the ORFs *SCO3638/39*, *SCO2165/66* and *SCO3640/41* in any of the conditions tested. The mutant Δ*SCO1744/45* showed a pleiotropic phenotype. The ACT, RED, and CDA productions on some media were triggered, suggesting a negative role of this system in the antibiotic production. In addition, the morphological differentiation was accelerated. An opposite pleiotropic phenotype was revealed for the *ΔSCO4596/97/98* mutant (TCS formed by two HKs and one RR). This mutant showed a decrease in ACT, RED, and CDA antibiotic productions and a delay in differentiation, which indicates that this system is a positive global regulator of the antibiotic production and differentiation. Microarray analyses of the *ΔSCO4596/97/98* and wild-type transcriptomes were performed.

## Results

### Construction of null mutant strains

According to the annotations of the *S. coelicolor* database genome (http://strepdb.streptomyces.org.uk/ [22]), five TCSs were selected. Four of them, SCO1744/45, SCO2165/66, SCO3638/39, and SCO3640/41, shared about 30% identity between their corresponding HKs and that of the well-known global antibiotic regulator *absA1/A2*, AbsA1. Additionally 50% identity was found between their RRs and the AbsA2 regulator, which are considered to be homologues (Table S1 and Table S2). The fifth one, composed of two HKs (SCO4597 and SCO4598, which share 57% identity) and one RR (SCO4596), presented less similarity to *absA1/A2* (25% HKs-AbsA1 and 33% RR-AbsA2) but both HKs were predicted to be functionally associated to AbsA1 using STRING application (*S*earch *T*ool for the *R*etrieval of *I*nteracting *G*enes/Proteins) (http://string.embl.de/newstring_cgi/show_input_page.pl) [23] (AbsA1-SCO4598 association score of 0.726 just below the AbsA1–AbsA2 and AbsA1-RedZ scores, 0.949 and 0.923, respectively; AbsA1-SCO4597 association score of 0.691). To determine the relevance of these five TCSs in antibiotic production, null mutant strains of each system were obtained from the *S. coelicolor* M145 strain by the REDIRECT procedure (see Material and Methods). The correct replacement of the genes by the cassette was confirmed by Southern blot hybridization using appropriate DNA probes (data not shown).

To detect putative alterations in antibiotic production and/or development of cells in the mutant strains compared to the wild type, all of them were grown on several solid media at 30°C. The media used were a minimal medium (NMMP) and different complex media (NA, YEPD, R2YE, PGA and MSA).

**Figure 1:**
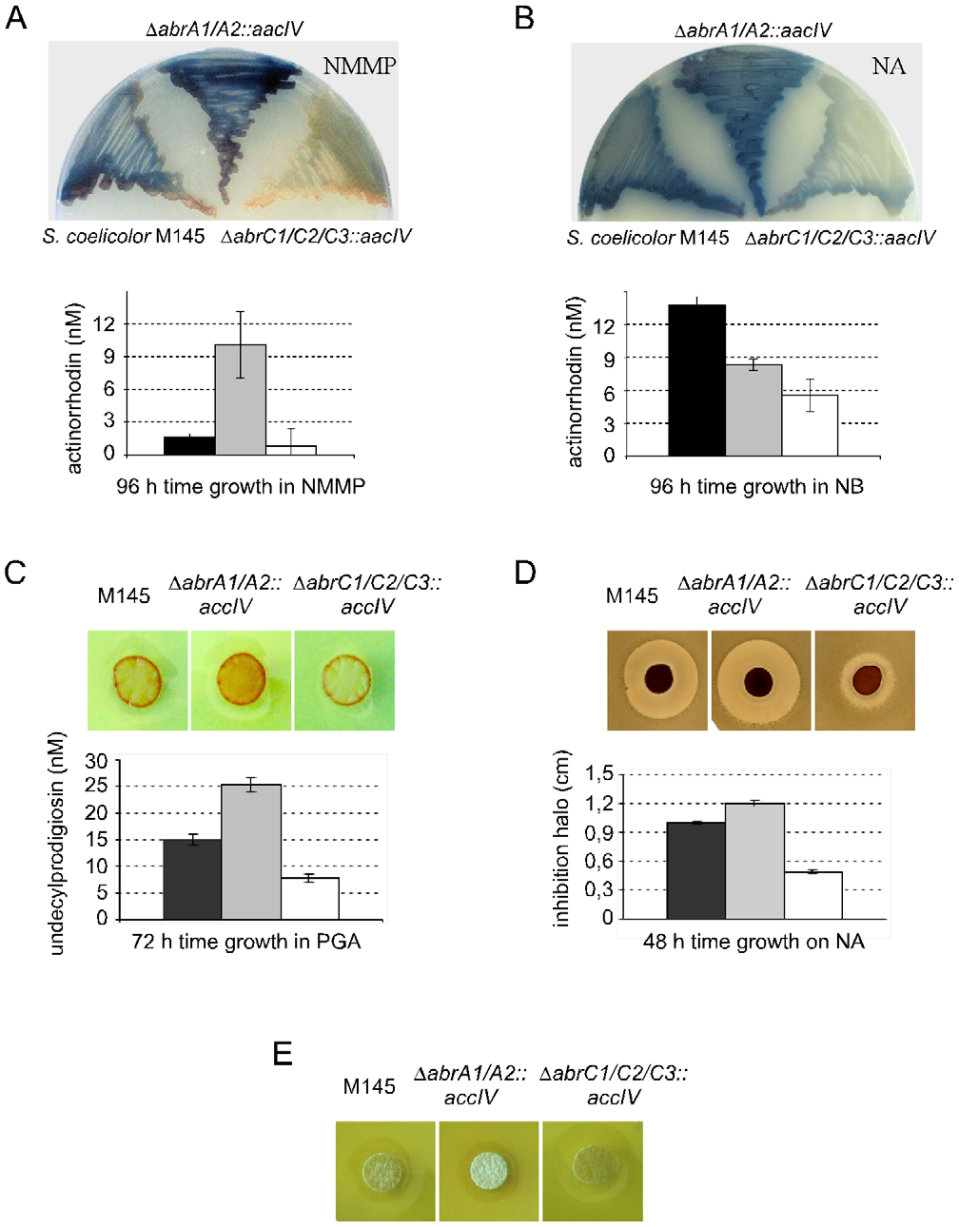
Antibiotic production and differentiation of the different strains. Wild-type strain: *S. coelicolor* M145. Mutant strains: *S. coelicolor ΔabrA1/A2* and *S. coelicolo*r *ΔabrC1/C2/C3*. A: ACT production on NMMP solid (top) and liquid (bottom) medium; B: ACT production on NA solid (top) and NB liquid (bottom) medium; C: RED production on PGA solid (top) and liquid (bottom) medium; D: CDA production bioassay against *B. subtilis* on NA solid medium (top) and inhibition halo diameter quantification (bottom); E: differentiation assay on YEPD (two days' growth). *S. coelicolor* M145 (black columns), *ΔabrA1/A2::aacIV* (grey columns), *ΔabrC1/C2/C3::aacIV* (white columns). Error bars correspond to standard deviation of four independent experiments.

Two of the five TCSs selected null mutant strains (*ΔSCO1744/45::accIV* and *ΔSCO4596/97/98::accIV*) consistently displayed significant differences in antibiotic production and differentiation compared to the wild type (Figure 1). No differences were observed in any conditions for the mutants of the systems *SCO3638/39* and *SCO3640/41* (data not shown). The absence of the system *SCO2165/66* in the mutant seemed to slightly increase production of the three antibiotics (ACT, RED, and CDA) in R2YE, PGA, and NA media respectively but these results were difficult to replicate and need further study (data not shown).

Clearly, the effect of mutations *ΔSCO1744/45::accIV* and *ΔSCO4596/97/98::accIV* was medium-dependent, especially in the production of ACT. Although differences could also be seen on R2YE and YEPD media (data not shown), the strongest effects in ACT production were on NMMP and on NA (Figure 1A, 1B). Both mutants showed different phenotypes on both solid media: the mutant Δ*SCO1744/45::accIV* displayed an ACT overproduction after three days' growth, while *ΔSCO4596/97/98::accIV* strain produced significantly less of this antibiotic molecule compared to the wild type (Figure 1A, 1B). To quantify these observations, liquid cultures were performed determining the rate growth and ACT production of each strain in both media at different times. As shown in Figure 1A (lower panel), the ACT production in the mutant Δ*SCO1744/45::accIV* in liquid NMMP was increased more than sixfold compared to the wild type at 96 h and the production in the mutant *ΔSCO4596/97/98::accIV* was about the half of the wild type. On the other hand, ACT production of *ΔSCO4596/97/98::accIV* strain in NB was about 40% of that of the wild type but less production of ACT in the mutant Δ*SCO1744/45::accIV* in NB was also observed showing a more complex nutritional behaviour of this mutant. The growth rates of the strains were similar in both liquid media (Figure S1).

Differences in undecylprodigiosin production were also observed on R2YE but mainly on PGA solid media (Figure 1C). Quantification of RED production in PG liquid medium showed that Δ*SCO1744/45::accIV* produces 67% more and *ΔSCO4596/97/98::accIV* approximately 50% less compared to the wild-type strain. As mentioned, this is not due to a growth defect because the growth curves of the three strains were almost identical in the culture conditions used (Figure S1).

CDA production, measured as the inhibition halo against *Bacillus subtilis*, was evaluated on NA plates in the presence or absence of calcium (see Materials and Methods) (Figure 1D). Once again, Δ*SCO1744/45::accIV* had higher CDA production than the wild-type strain (8.5%, the average of seven independent assays), and *ΔSCO4596/97/98::accIV* presented a decrease of 32% of the inhibition halo.

Finally, YEPD was the medium used to document the morphological development. Δ*SCO1744/45::accIV* mutant presented an accelerated formation of aerial mycelium, while *ΔSCO4596/97/98::accIV* showed a clear delay in the differentiation in these culture conditions (Figure 1E).

In summary, mutant *ΔSCO1744/45::accIV* overproduced the three antibiotics and also showed a positive role in differentiation (the aerial mycelia and spores appeared sooner than in the wild-type strain). In contrast, strain *ΔSCO4596/97/98::accIV* showed a decreased production of the antibiotics ACT, CDA, and RED, and the differentiation was delayed.

From these results we can conclude that the two-component systems composed by the ORFs: *SCO1744/45* and *SCO4596/97/98*, acted as ***a***nti***b***iotic production ***r***egulators, and thus they were called *abrA1/A2*, and *abrC1/C2/C3*, respectively.

### Genetic complementation of TCSs null mutants

To make sure that the null mutant phenotypes observed were due to the absence of TCSs genes and not to mutagenesis polar effects, the genetic complementation was carried out. First of all, the mutagenesis apramycin cassette of the each null mutant strain (*ΔabrA1/A2::accIV* and *ΔabrC1/C2/C3::accIV*) was eliminated to avoid possible polar effects (see Materials and Methods). The resulting strains harboured a small scar (83 bp) in place of the former antibiotic resistance sequence (*ΔabrA1/A2* and *ΔabrC1/C2/C3*) and displayed the same phenotypes as the original mutants (data not shown).

**Figure 2:**
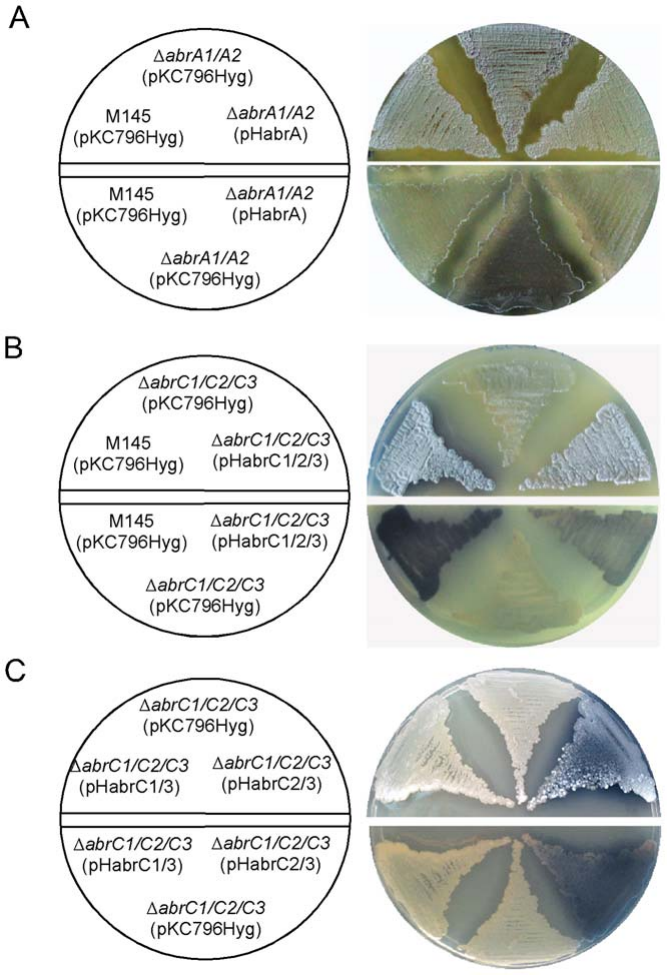
Mutant complementation. A: Complementation of *ΔabrA1/A2* phenotypes by the integrative plasmid pHabrA derived from pKC796Hyg on NMMP. Top: morphological differentiation. Bottom: ACT production. B: Complementation of *ΔabrC1/C2/C3* phenotypes by the integrative plasmid pHabrC1/2/3 derived from pKC796Hyg on NA (2 days). Top: morphological differentiation. Bottom: ACT production. C: Complementation of *ΔabrC1/C2/C3* phenotypes by the integrative plasmid pHabrC1/3 and pHabrC2/3 derived from pKC796Hyg on NA (3 days). Top: morphological differentiation. Bottom: ACT production.

The reverting strains were obtained by ectopic integration of plasmids derived from pKC796Hyg in the ΦC31 attachment site: pHabrA (whole system), pHabrC1/2/3 (whole system), pHabrC1/3 (with a deletion in the gene encoding kinase AbrC2), and pHabrC2/3 (with a deletion in the gene encoding kinase AbrC1) (see Materials and Methods). Wild type and mutant strains with the integrated pKC796Hyg plasmid were used as controls. It is worth mentioning that integration of any plasmid in the ΦC31 site provokes a decrease in the antibiotic production [24], especially on NMMP medium. As shown in Figure 2, both *ΔabrA1/A2* (pHabrA) and *ΔabrC1/C2/C3* (pHabrC1/2/3) restored the phenotypes of ACT production and differentiation of wt (pKC796Hyg), although partially in the case of ACT production in *ΔabrC1/C2/C3* (pHabrC1/2/3) strain (Figure 2B). The *ΔabrC1/C2/C3* mutant phenotype could also be reverted by complementation with pHabrC2/3 but not with pHabrC1/3 (Figure 2C) suggesting a more important role of HK AbrC2 (SCO4597) in the signalling network in this medium.

The reversion of the mutant phenotypes was also analysed using multicopy plasmids derived from pN702GEM3 (high copy number: 40–100 copies/genome) harbouring either *abrA1/A2* (plasmid pNXabrA) or *abrC1/C2/C3* genes (plasmid pNabrC) (see Materials and Methods). When *abrA1/A2* genes were expressed in the multicopy plasmid the mutant phenotype was not only reverted (Figure S2), but also antibiotic production (ACT, RED and CDA) was even lower than in the wt (pN702GEM3). Additionally, the strain Δ*abrA1/A2* (pN702GEM3) had an accelerated aerial mycelium formation, as opposed to Δ*abrA1/A2* (pNXabrA) and wt (pN702GEM3) strains.

Unexpectedly, the Δ*abrC1/C2/C3* (pNabrC) strain had even less antibiotic production (ACT, RED, and CDA) than the mutant Δ*abrC1/C2/C3* (pN702GEM3) strain (Figure S3). However, when the genes were expressed from a low copy number plasmid pAbrC (derived from pHJL401 5–10 copies/genome see Materials and Methods), both phenotypes, antibiotics production and morphological differentiation, were reverted (Figure S3).

Our results confirm that both systems have different roles in regulation; while both affect antibiotic production and morphological differentiation pathways, the AbrA1/A2 is a negative pleiotropic regulator and AbrC1/C2/C3 is a positive pleiotropic regulator.

### Microarray analysis of the *ΔabrC1/C2/C3* strain

**Figure 3:**
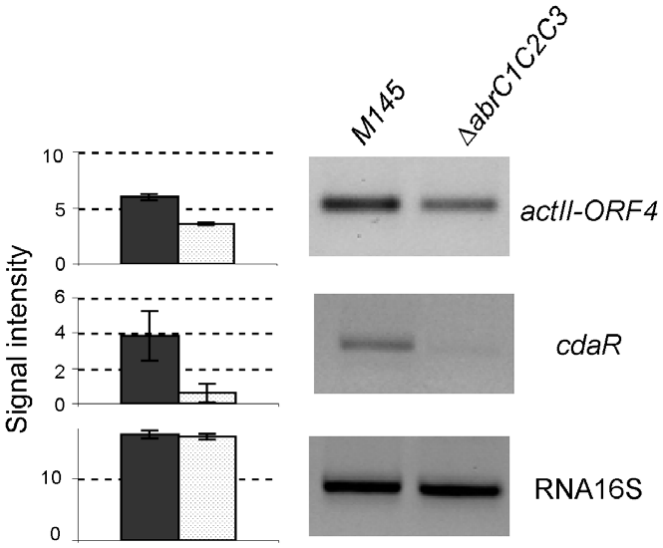
RT-PCR assays. *S. coelicolor* M145 and *ΔabrC1/C2/C3* RNAm amplification of *actIIORF4* and RNA 16S (25 cycles) and *cdaR* (40 cycles) by RT-PCR. Ribosomal RNA 16S amplification (25 cycles) was used as control. Quantification of signal intensities is shown at the left.

In order to determine the genes whose expression could be affected by the lack of the three-component system, microarrays assays comparing gene expression levels between *ΔabrC1/C2/C3* and wild-type strains were performed. Total RNA preparations were obtained from cultures (four replicates) grown for 50 h on NA solid medium (see Materials and Methods). Statistical analysis of the microarray results using *limma* provided a differential expression value and an associated *p*-value for each gene. After correction of these *p*-values for multiple testing (FDR or *pdf*, see Materials and Methods), only a few genes were statistically significant (*p*<0.05) (see Table 1). Most of them, however, encoded either hypothetical proteins or proteins of putative functions, which were not easily correlated with the phenotype observed. If uncorrected *p*-values were considered (*p*<0.05), 201 genes appeared to be upregulated and 202 genes downregulated in the mutant strain. This set of genes should be taken with caution since it might contain false positives. Nevertheless, certain genes showed expression changes that could be correlated with phenotypic observations or with a shared function. Thus, the lower antibiotic production of the mutant Δ*abrC1/C2/C3* was reflected in the expression changes of structural and regulatory genes. Particularly, the ACT and CDA pathway-specific regulators *actIIORF4* and *cdaR* were slightly downregulated (see Table 1). Semiquantitative RT-PCR (see Materials and Methods) confirmed this (see Materials and Methods). When compared to the wild-type strain M145 the transcript levels of these genes in the mutant strain decreased to 60% and 16%, respectively (Figure 3).

**Table 1:** Selected genes differentially expressed in the Microarray assay *ΔabrC1/C2/C3 vs* M145 by their p-value<0.05 and FDR/pfp<0.05 or their biological meaning (italics).

	SCO/name	Function	Fold change	p-value	p-value FDR/pfp
**Upregulated**	0122	Putative flavin containing monooxygenase	2.31	0.022	0.038
	0277	Hypothetical protein	2.83	0.0012	0.0225
	0850	Putative membrane protein	3.23	0.0003	<0.001
	3309	Hypothetical protein	2.26	0.048	0.030
	6259	Probable ABC sugar transport	2.96	0.0178	0.040
	6557	Putative neuramidase	2.32	0.0029	0.030
	*5627*	*Ribosome recycling factor*	*1.8*	*0.0003*	*0.232*
**Downregulated**	4559	Putative electrontransfer oxidoreductase	−2.88	0.0053	<0.001
	4612	Putative amino acid transporter	−2.57	0.0096	0.02
	*5085/actIIORF4*	*Actinorhodin cluster activator protein*	*−1.3*	*0.048*	*0.897*
	*5089/actIORF3*	*Actinorhodin polyketide synthase acyl carrier protein*	*−1,72*	*0,0017*	*0.325*
	*3217/cdaR*	*Transcriptional activator protein*	*−1.4*	*0.0009*	*0.777*
	*0708*	*Putative branched-chain amino acid transport protein*	*−1.63*	*0.027*	*0.332*
	*1599/rpmI*	*50S ribosomal protein*	*−1.8*	*0.0036*	*0.333*
	*1998/rpsA*	*30S ribosomal protein*	*−1.65*	*0.0252*	*0.357*
	*2563/rpsT*	*30s ribosomal protein*	*−1.78*	*0.0023*	*0.322*
	*2596/rpmA*	*50S ribosomal protein*	*−1.6*	*0.0412*	*0.407*
	*3023*	*Adenosylhomocysteinase*	*−1.5*	*0.0004*	*0.545*
	*3427/rpmE*	*Putative 50S ribosomal protein*	*−2.19*	*0.012*	*0.180*
	*3430/rpsN*	*Putative 30S ribosomal protein*	*−1.73*	*0.0074*	*0.357*
	*3908/rpsR*	*Putative 30S ribosomal protein*	*−2*	*0.01*	*0.220*
	*4661/fusA*	*Elongation factor G*	*−1.8*	*0.0039*	*0.273*
	*4703/rplD*	*50S ribosomal protein*	*−1.82*	*0.04*	*0.213*
	*4718/rplR*	*50S ribosomal protein*	*−1,6*	*0.037*	*0.453*
	*4721/rplO*	*50S ribosomal protein*	*−1.62*	*0.045*	*0.386*
	*5591/rpsP*	*30S ribosomal protein*	*−1.75*	*0,029*	*0.333*

Therefore, the downregulation of the mentioned SARPs encoding genes causes, at least partially, a decrease in ACT and CDA production in Δ*abrC1/C2/C3* as indeed the phenotypic assays showed.

Expression differences in translation-related genes were also found (Table 1). Some genes encoding ribosomal proteins and amino acid transporters proteins were downregulated in the mutant strain, while the ribosomal recycling factor encoding gene (*frr*, *SCO5627*) was upregulated.

## Discussion

In this paper, we reported the study of five new TCSs from *S. coelicolor* M145 and the involvement of two of them, named AbrA1/A2 and AbrC1/C2/C3, in antibiotic production. Notoriously, the phenotype of both knockout strains was conditional. This fact is not surprising since the TCSs are frequently aimed to respond to specific environmental signals (i.e. AfsQ1-Q2-sigQ [25]), which can be easily missed in some culture media or conditions.

Additionally, our data show how both TCS systems studied played pleiotropic roles in bacteria since not only affected different antibiotic pathways but also different biological processes such as morphological differentiation. Up to date most of the characterized TCSs in *S. coelicolor* have been reported to have an effect on antibiotic production (i.e. CutR/S [26], EcrA1/A2 [27], PhoR/P [28], AbsA1/A2 [18], RapA1/A2 [29]). However, just one among them, (AfsQ1-Q2-sigQ) has been described to be involved in both secondary metabolism and morphological development [25].

As detailed in the results section, the null mutant strain *ΔabrA1/A2* (SCO1744/45) overproduced the three antibiotics tested in a medium dependent manner. This fact makes this system extremely interesting since it could be used to overproduce clinical useful antibiotics by expressing abrA1/A2 alleles in heterologous streptomycetes as has been recently reported for the system AbsA1/A2 [30]. Interestingly, this system only has an orthologue in *S.lividans* being absent in all the other *Streptomyces* species sequenced to date. However, the *S. lividans* knockout does affect neither antibiotic production nor morphological differentiation (data not shown). Therefore, this system seems to represent a *S. coelicolor* specific antibiotic regulator.

The system, AbrC1/C2/C3, must be considered special because it has two kinases and one regulator. Besides, each gene is separated from the upstream ORF by a DNA sequence long enough to have its own promoter (286, 112, and 171 nt, respectively). Therefore, each gene might be expressed independently in order to suit its own needs. This system is conserved in all the *Streptomyces* species sequenced so far as well as in the ones those are in the process of being sequenced. Furthermore, the response regulator protein SCO4596 shares about 80% identity at the amino acid level in all the species. This consistently indicates an important role for this special system.

Our data demonstrate that the deletion of the three genes originates a strain with reduced capacity to produce the three antibiotics studied, ACT, RED, and CDA. Similar phenotypes were obtained with the expression of these three genes in a high copy number plasmid but not in a low copy number where the phenotypes were reverted to the wild type ones (Figure S3), showing that this effect was dose dependent. On the contrary, the mutant phenotype with respect to morphological differentiation was reverted even in multicopy number plasmid (Figures 2 and S3). This suggests that separate mechanisms underlie the effects of AbrC1/C2/C3 on antibiotic production and differentiation, as was found with AbsB protein [31].

Microarray analysis and RT-PCR studies demonstrated the role of AbrC1/C2/C3 over antibiotic production was at least partly through transcription of pathway-specific regulator genes *actIIORF4* and *cdaR*. However, with the data obtained to date, we cannot determine whether this is a direct regulation due to the binding of AbrC3 to the specific promoters of the pathway regulators or an indirect effect through a complex regulatory network. Therefore, deeper studies will be performed to understand the role of this TCS in the regulation of antibiotic production in the pigmented streptomycete *S. coelicolor*. Expression differences between *ΔabrC1/C2/C3* mutant and wild-type strains have also been found in genes associated with translation machinery. We hypothesized that a lower expression of some ribosomal protein genes (9 out of 62) in the mutant may affect the synthesis of proteins needed for the production of antibiotics, and in response cells try to compensate this by increasing the ribosomal recycling factor. The relation between enhanced protein synthesis during the stationary phase and the expression of regulatory proteins governing antibiotic production has been suggested previously [32], [33]. In addition, previous work has correlated the ribosomal proteins and the *frr* overexpression with ACT production [34] and more recently with avermectin overproduction [35].

It is widespread known that antibiotic production in *S. coelicolor* is a complex process that is regulated by a broad network of genes. In this paper two new two-component global regulators in this network have been identified. It is noteworthy that, they are among the very few TCSs identified on *S. coelicolor* that are affecting two different but related processes: the antibiotic production and developmental differentiation. One, *abrA1/A2*, is a negative regulator; the other, *abrC1/C2/C3*, a three-component system composed by two HKs and one RR, is a positive regulator.

## Materials and Methods

### Strains, media and culture conditions

*Escherichia coli* strains growth was accomplished as described previously [36]. BW25113 (pIJ790) (containing the λRed system) is an *E. coli* K12 (*ΔaraBAD*, *ΔrhaBAD*) derivative [37]; non-methylating ET12567 (pUZ8002) is *dam*, *dcm*, *hsdS*, *cat*, *tet* containing the *atra* genes [38] and *E. coli* DH5α (pBT30) is *recA*, *cat*, *bla* containing *flp* gene [37]. For CDA bioassays a wild-type strain of *Bacillus subtilis* (CECT 4522) was grown as an overlay on NA medium. *S. coelicolor* M145 (prototroph, SCP1^−^, SCP2^−^, methylenomycin^−^) and its mutant strain derivatives were grown on R2YE, NA, MSA, PGA, YEPD, and NMMP [39]. Liquid cultures were performed in 100 ml baffled flasks with 15 ml medium each. When necessary, the medium was supplemented with antibiotics (*E.coli* media: 100 µg ml^−1^ for ampicillin, 50 µg ml^−1^ for apramycin, 50 µg ml^−1^ for kanamycin, 34 µg ml^−1^ for chloramphenicol, and 25 µg ml^−1^ for nalidixic acid. *S. coelicolor* media: 20 µg ml^−1^ for neomycin and 20 µg ml^−1^ for hygromycin).

### Isolation and manipulation of DNA

Plasmid isolation, restriction enzyme digestion, ligation, and transformation of *E. coli* and *S. coelicolor* were carried out by methods of Sambrook *et al* [40] and Kieser *et al* [39], respectively. The plasmids and cosmids used are listed in Table 2. Total genomic DNA from *S. coelicolor* (gDNA) was isolated from a 24–36 h cultures in TSB medium following the procedure described in Hopwood *et al* [41], but scaled to 1–2 g of mycelium.

**Table 2:** Plasmids and cosmids used in this work.

Vector	Characteristics	Reference
pIJ790	λ-RED (*gam*, *beta*, *exo*), *cat*, *araC*, *rep101^ts^*	[42]
pIJ773	*aac(3)IV* (Apra^R^)+*oriT*, FRT sites	[42]
pUZ8002	*tra*, *neo*, RP4	[51]
SCI11	Supercos-1 derivative: *bla*, *neo*. Contains genes *SCO1744/45*	[52]
SC5F7	Supercos-1 derivative: *bla*, *neo*. Contains genes *SCO2165/66*	[52]
SCH10	Supercos-1 derivative: *bla*, *neo*. Contains genes *SCO3638/39/40/41*	[52]
SCD20	Supercos-1 derivative: *bla*, *neo*. Contains genes *SCO4596/97/98*	[52]
ΔSCI11-1	SCI11 *ΔSCO1744/45::aac(3)IV*	This work
ΔSC5F7-1	SC5F7 *ΔSCO2165/66::aac(3)IV*	This work
ΔSCH10-1	SCH10 *ΔSCO3638/39::aac(3)IV*	This work
ΔSCH10-2	SCH10-2 *ΔSCO3640/41::aac(3)IV*	This work
ΔSCD20-1	SCD20 *ΔSCO4596/97/98::aac(3)IV*	This work
ΔSCD20-1 SCAR	SCD20 *ΔSCO4596/97/98*	This work
ΔSCI11-1 SCAR	SCI11 *ΔSCO1744/45*	This work
pXHis1	*E. coli* plasmid Amp resistance	[45]
pAY001	pXHis1 derivative containing promoter region of *SCO1744/45*	This work
pAY002	pAY001 derivative containing *SCO1744/45*	This work
pN702GEM3	*E. coli–Streptomyces* shuttle vector; Neo/Kan resistance	[45]
pNXabrA	pN702GEM3 derivative containing *SCO1744/45*	This work
pNabrC	pN702GEM3 derivative containing *SCO4596/97/98*	This work
pHJL401	*E. coli–Streptomyces* shuttle vector; Amp/Tsr resistance	[46]
pAbrC	pHJL401 derivative containing *SCO4596/97/98*	This work
pKC796Hyg	Integrative plasmid with hygromycin resistance	[43]
pHabrA	pKC796Hyg derivative containing *SCO1744/45*	This work
pHabrC1/2/3	pKC796Hyg derivative containing *SCO4596/97/98*	This work
pHabrC1/3	pKC796Hyg derivative containing *SCO4596/98*	This work
pHabrC2/3	pKC796Hyg derivative containing *SCO4596/97*	This work

### Deletion of the TCSs selected

REDIRECT PCR-targeting technology [42] was used to replace the genes of the entire coding region of each TCS (comprising histidine kinase and response regulator) to an apramycin (*aac(3)IV* gene) resistance cassette. Mutagenic cassettes were flanked by the recognition sequence of *E. coli* Flipase (FRT) and contained the conjugation transfer origin *oriT* (FRT-*aac(3)IV-oriT*-FRT) and were amplified using the High-Fidelity Expand PCR system (Roche Co.) with the primers listed in Table S3 using plasmid pIJ773 as template. The generated cassettes were introduced into *E. coli* BW25113 (pIJ790) harbouring the appropriate cosmid for each studied system (Table 2: SCI11, SC5F7, SCH10 and SCD20; http://strepdb.streptomyces.org.uk/ [22]) and preinduced for λRed functions, by the addition of arabinose, to obtain a target gene-disrupted version of the mutant cosmids. The disrupted cosmids, confirmed by restriction analysis, were isolated and transferred from *E. coli* ET12567 (pUZ8002) to *S. coelicolor* M145 by conjugation. Exconjugants were selected on MSA medium containing apramycin (50 µg ml^−1^), and the double crossover products identified by screening their sensitivity to kanamycin (50 µg ml^−1^). The disruptions were confirmed by Southern hybridization and the DIG DNA labelling and detection kit (Roche Co.) was used for probe preparation (obtained with primers of Table S3).

To avoid putative polar effects of the mutagenesis cassette gene replacement in *S. coelicolor* M145, the antibiotic resistant marker and the *oriT* region were eliminated in two steps. In a first step, the corresponding disrupting cosmids were introduced in *E. coli* DH5α (pBT30) strain (harbouring the Flipase gene, FLP) in which, the recombination between both FRT mutagenesis cassette-flanking regions takes place. In these new cosmids only 81 base pairs (SCAR) remained in frame with the adjacent ORFs. Afterwards, in the second step, the SCAR cosmids were transferred to the *Streptomyces* apramycin-resistance mutant strains by protoplast transformation, selecting neomycin-resistance clones in the first place (unique recombination). Finally the strains were apramycin and neomycin-sensitive (double recombination). PCR assays confirmed the correct recombination in the new *Streptomyces* mutant strains.

### Plasmid constructions

All the plasmids used in this work are listed in Table 2. Integrative plasmid pHabrA was obtained by cloning PCR-amplified *abrA1/A2* genes and their own promoter in the shuttle *Streptomyces* integrative plasmid pKC796Hyg [43]. In the intermediate pAY001 plasmid, the promoter PCR fragment was amplified with primers AY-033 (adding an EcoRI site), and AY-034 (adding an NdeI site) (Table S3), using SCI11 as a template, was cloned in pXHis1 plasmid [44]. pAY002 derivative plasmid harbours the pair of genes amplified by PCR with primers AY-035 (additional NdeI site) and AY-036 (adding an XhoI site) in the NdeI/XhoI sites of pAY001 plasmid. Fragment BglII/BglII from pAY002 was finally cloned in pKC796Hyg plasmid, yielding pHabrA plasmid.

pNXabrA plasmid was obtained by cloning the fragment NdeI/HindIII from pAY002 plasmid in the sites of the pNX24 plasmid (pN702GEM3 derivative [45]). In this shuttled (*E.coli-Streptomyces*) multicopy plasmid the xylanase promoter *xys*Ap controls *abrA1/A2* gene expression.

The three genes *abrC1/C2/C3*, and their intergenic regions were cloned in a pN702GEM3 plasmid yielding a multicopy plasmid in several steps. An intermediate *E. coli* monofunctional plasmid called pSCD20 was constructed by cloning a BspEI/PmII fragment from a subclone of SCD20 cosmid in the BspEI/Ecl136II sites of pHJL401 [46]. Afterwards, the fragment EcoRV/HindIII from pSCD20 was introduced in pN702GEM3 to get pNabrC. The low copy number pAbrC plasmid derived from pHJL401 [46] was obtained by cloning the BglII/HindIII fragment from pNabrC in the BamHI/HindIII sites of pHJL401.

To obtain the integrative plasmid for mutant Δ*abrC1/C2/C3* complementation, pNSCD20 was digested with HindIII, filled with Klenow polymerase, and BglII digested. This fragment was inserted into the BlgII/EcoRV sites of pKC796Hyg to get pHabrC1/2/3. Plasmid with *abrC1* gene disrupted was got by digesting pHabrC1/2/3 with XhoI and religated (eliminating a fragment of 260 nt containing the promoter and the 5′ end of the gene), yielding pHabrC2/3. To disrupt *abrC2* gene an inner fragment of 1180 nt was eliminated from pHabrC1/2/3 using SfiI/AgeI sites and by treatment with T4 DNA polymerase before ligation, the plasmid got was named pHabrC1/3.

The new plasmids were introduced into the corresponding strains by protoplast transformation as previously described [39].

### Antibiotic determination

Antibiotic production was assayed on solid media as described below. Plates were inoculated with 10^3^ spores streaked or added to a 5 µl drop. For CDA production the strains were grown on NA medium at 30°C for 2 days. Afterwards, the plates were overlaid with 5 ml of soft agar plus 60 mM Ca(NO_3_)_2_ inoculated with *B. subtilis* as the test microorganism (0.2 ml, 0.25 DO) and incubated at 30°C for 20 h. A replica plate without calcium was used as a negative control. For ACT production on solid media, the strains were grown on different media (YEPD, R2YE, NMMP, NA) at 30°C for at least 3 days to observe the blue halo around the colonies. RED production was detected on PGA medium after 2 days as the red colour of colonies.

The ACT and RED antibiotic productions were quantified in liquid cultures using the standard spectrophotometric method [39] with minor modifications. 15 ml of medium were inoculated with 4×10^6^ spores/ml. Culture samples were mixed with 1N KOH overnight at 4°C, centrifuged (15000 g, 10 min), and A_640_ of supernatants were determined to quantify ACT (ε_640_ = 25320). To quantify RED, pellets were washed twice in 0.5 M HCl and extracted in 0.5 M HCl-methanol for 2 h. After centrifugation (15000 g, 5 min), supernatant' absorbance were measured (ε_530_ = 100500). Dry weight of samples at different times was measured to monitor culture growth.

### Microarrays assays

For RNA extraction from *S. coelicolor* wild-type and Δ*abrC1/C2/C3* mutant strains, NA plates covered with a cellophane sheet were inoculated with 7.5×10^6^ spores and incubated at 30°C for 50 h. Prior to RNA isolation using a RNeasy Midi Kit (Quiagen) the mycelia was harvested and suspended in RNA-protect Bacteria Reagent (Qiagen). An additional step with RNase free DNase (Qiagen) was incorporated to remove any contaminating DNA. The quality and concentration of RNA were assayed using gel electrophoresis and spectrophotometer assays (Q-bit and Agilent bioanalizer). Four biological replicates were used.

cDNA *versus* gDNA microarrays experiments were chosen due to the advantages described elsewhere [47], [48]. The *S. coelicolor* SCo40 microarrays used were obtained from the Functional Genomics Laboratory of Surrey University (UK) [49]. The Pronto! Universal Microarray Hybridization kit (Corning, # 40026) was used for pretreatment and prehybridization. Cy3-cDNA and Cy5-gDNA labelling reactions were performed according to the recommendations described by http://www.surrey.ac.uk/SBMS/Fgenomics [49]. Hybridization assays were done as in Rodríguez-García *et al*. [28] and TIFF images were generated by Genepix DNA Microarray Scanner 4000B and processed with Genepix Pro 4.0 software. Bioconductor software package limma (*l*inear *m*odels for *m*icroarray *a*nalysis) and rank products were used to analyse and assess the statistical significance of the data [28], [50]. Background correction was applied using the normexp function. Then, the log of Cy3/Cy5 intensities were normalized using block-weighted medians and global loess. The different *p*-values of the contrast between both strains were corrected for multiple testing FDR (*f*alse *d*iscovery *r*ate) or by the rank products *pfp* method (*p*roportion of *f*alse *p*ositives). To consider a gene differentially expressed, it should have passed at least one of these criteria: limma FDR-corrected *p*-value<0.05 or rank products *pfp* value<0.05. All data is MIAME compliant and the raw data has been deposited in a MIAME compliant database (ArrayExpress, accession number E-MEXP-2841)

### Semiquantitative RT-PCRs

RT-PCR assays were performed with 200 ng RNA in a final volume of 20 µl with the Superscript™ One-Step RT-PCR with Platinum® Taq System Kit (Invitrogen). The primers used are specified in Table S3. Reactions were made as follows: 30 min at 55°C (cDNA synthesis); 2 min at 95°C; 20–40 cycles: 45 sec at 94°C, 30 sec at 65°C and 40 sec at 65°C; 10 min at 72°C. To check the DNA absence in the RNA samples, similar reactions avoiding the cDNA synthesis step were done in parallel. 2 µl of each reaction were run in 1.6% agarose gel buffered with TAE 1×. Each set of reactions was repeated varying the number of cycles to ensure that the PCR had not reached the plateau phase. As a positive internal control RT-PCR of 16S RNA was used. RT-PCR band images were quantified using Quantity One Analysis software 4.6.6 (Bio-Rad).

## Supporting Information

Table S1 **Identity percentages among the sensor kinases by a local alignment (Emboss).**
(DOC)

Table S2 **Identity percentages among the response regulators by a local alignment (Emboss).**
(DOC)

Table S3 **Primers used in this work.**
(DOC)

Figure S1 **Growth curves of the different strains** in NMMP (A), NB (B) and PGA (C) *S. coelicolor* M145 (triangles), *S. coelicolor ΔabrA1/A2* (circles) and *S. coelicolor ΔabrC1/C2/C3* (squares). Error bars correspond to standard deviation of two independent experiments measured by duplicate.
(TIF)

Figure S2 **Phenotypes of strains expressing abrA1/A2 in multicopy plasmid.** A: Effect of the expression of *abrA1/A2* genes by the high copy number plasmid pNXabrA derived from pN702GEM3 on NMMP medium. Top: morphological differentiation. Bottom: ACT production. B: CDA bioassays on NA medium, RED production on PGA medium, and morphological differentiation on YEPD medium (2 days), in the different strains.
(TIF)

Figure S3 **Phenotypes of strains expressing abrC1/C2/C3 in multicopy plasmids.** A: Effect of expression of *abrC1/C2/C3* genes by the high copy number plasmid pNabrC derived from pN702GEM3: ACT production on NA medium, CDA bioassays on NA medium, RED production on PGA medium, and MD morphological differentiation on YEPD medium (2 days), by the different strains. B: Effect of expression of *abrC1/C2/C3* genes by the low copy number pAbrC plasmid derived from pHJL401: ACT production on NA medium, CDA bioassays on NA medium, RED production on PGA medium, and MD morphological differentiation on YEPD medium (3 days), by the different strains.
(TIF)
