# Supplementary material for: Novel Two-Component Systems Implied in Antibiotic Production in Streptomyces coelicolor
Source: PLoS One. 2011 May 20;6(5):e19980. doi: 10.1371/journal.pone.0019980 (PMC3098853; doi:10.1371/journal.pone.0019980)
Supplement: Table S1 — Identity percentages among the sensor kinases by a local alignment (Emboss). (DOC) [file pone.0019980.s001.doc]

### Table S1 – Identity percentages among the sensor kinases by a local alignment (Emboss)

|  | ***SCO1744*** | ***SCO2166*** | ***SCO3639*** | ***SCO3641*** | ***SCO4597*** | ***SCO4598*** | ***AbsA1*** |
| --- | --- | --- | --- | --- | --- | --- | --- |
| ***SCO1744*** | 100 |  |  |  |  |  |  |
| ***SCO2166*** | 34,6 | 100 |  |  |  |  |  |
| ***SCO3639*** | 33,2 | 34,7 | 100 |  |  |  |  |
| ***SCO3641*** | 35,2 | 36,6 | 44,3 | 100 |  |  |  |
| ***SCO4597*** | 25,4 | 26,8 | 25,5 | 25,6 | 100 |  |  |
| ***SCO4598*** | 26,9 | 25,3 | 22,9 | 24,3 | 57 | 100 |  |
| ***AbsA1*** | 30,7 | 29,9 | 28,9 | 30,6 | 24,6 | 22,8 | 100 |
