# Supplementary material for: Novel Two-Component Systems Implied in Antibiotic Production in Streptomyces coelicolor
Source: PLoS One. 2011 May 20;6(5):e19980. doi: 10.1371/journal.pone.0019980 (PMC3098853; doi:10.1371/journal.pone.0019980)
Supplement: Table S2 — Identity percentages among the response regulators by a local alignment (Emboss). (DOC) [file pone.0019980.s002.doc]

### Table S2 – Identity percentages among the response regulators by a local alignment (Emboss).

|  | ***SCO1745*** | ***SCO2165*** | ***SCO3638*** | ***SCO3640*** | ***SCO4596*** | ***AbsA2*** |
| --- | --- | --- | --- | --- | --- | --- |
| ***SCO1745*** | 100 |  |  |  |  |  |
| ***SCO2165*** | 51,8 | 100 |  |  |  |  |
| ***SCO3638*** | 57 | 52,2 | 100 |  |  |  |
| ***SCO3640*** | 60,1 | 53,8 | 75,7 | 100 |  |  |
| ***SCO4596*** | 35,9 | 35,2 | 36,5 | 37,4 | 100 |  |
| ***AbsA2*** | 43,1 | 44,6 | 48,1 | 46,6 | 33 | 100 |
