# Supplementary material for: Novel Two-Component Systems Implied in Antibiotic Production in Streptomyces coelicolor
Source: PLoS One. 2011 May 20;6(5):e19980. doi: 10.1371/journal.pone.0019980 (PMC3098853; doi:10.1371/journal.pone.0019980)
Supplement: Table S3 — Primers used in this work. (DOC) [file pone.0019980.s003.doc]

### Table S3 – Primers used in this work

| **Primers** | **Sequence (5´-3´)** | **Function** |
| --- | --- | --- |
| AY-001 | CGCGCCTACTCCCTCCGCAGTACACGCGGGCGGAGGTCATGTAGGCTGGAGCTGCTTC | Deletion cassette *SCO2165/66* (forward) |
| AY-002 | CATGGGAGACCCGTACTCCGGCTACCGTAAAAGACCGTGATTCCGGGGATCCGTCGACC | Deletion cassette *SCO2165/66* (reverse) |
| AY-005 | GGTCGCGGTCGTGGTCGAGCGACGGCGCGATCTGGCGTGATTCCGGGGATCCGTCGACC | Deletion cassette *SCO1744/45* (forward) |
| AY-006 | GAGCCCCCGCACACCGCGGCCACGGTGTGCGGGGGCTCATGTAGGCTGGAGCTGCTTC | Deletion cassette *SCO1744/45* (reverse) |
| AY-007 | CCCGGGTCGGTGCTTCCGAGGCATGGGCCGCCGGTGTCATGTAGGCTGGAGCTGCTTC | Deletion cassette *SCO4596/97/98* (forward) |
| AY-008 | TGGCGCGGCGTCGCGCCGCACGGCACGCTGACGTGTATGATTCCGGGGATCCGTCGACC | Deletion cassette *SCO4596/97/98* (reverse) |
| AY-009 | AGCGGCCGGTGACCGGTAGCGGCATCGCCCCCGCGGTCATGTAGGCTGGAGCTGCTTC | Deletion cassette *SCO3638/39* (forward) |
| AY-010 | TGGTGCGCCCCGCGGGCTACTGAGGAGCGACGCGCGGTGATTCCGGGGATCCGTCGACC | Deletion cassette *SCO3638/39* (reverse) |
| AY-011 | GTCTCAAGTACTACGGGGGCCACCGCGCGTCGCTCCTCATGTAGGCTGGAGCTGCTTC | Deletion cassette *SCO3640/41* (forward) |
| AY-012 | GCCCGCGGCGCCCGGCGACCACGGCAGGTGCACGCCATGATTCCGGGGATCCGTCGACC | Deletion cassette *SCO3640/3641* (reverse) |
| AY-033 | TTTTTTGAATTCCTCACGTCCTCCTGTGGGGGG | Amplification of *abrA1/A2* promoter (forward) |
| AY-34 | TTTTTTCATATGTCCGAAGCCTCCGGTGTGCC | Amplification of *abrA1/A2* promoter (reverse) |
| AY-035 | TTTTTTCATATGAACGACCTCTTCGGGCGGG | Amplification of *abrA1/A2* genes (forward) |
| AY-036 | TTTTTTCTCGAGTCAGCCCGTGCCGCCGGGG | Amplification of *abrA1/A2* genes (reverse) |
| AY-117 | TCAGGCGGCACGAGGTGGTGG | Forward RT-PCR amplification of RNA *actII-ORF4* |
| AY-118 | ACCCGGTGCTCCCCCAGCAG | Reverse RT-PCR amplification of RNA *actII-ORF4* |
| AY-059 | CCATCGAAGAGATCGGTCTTGAAC | Forward RT-PCR amplification of RNA *cdaR* |
| AY-060 | GCTGGCGCAGGAACTGGTGGAG | Reverse RT-PCR amplification of RNA *cdaR* |
| 16S F | TCACGGAGAGTTTGATCCTGGCTC | Forward RT-PCR amplification of RNA 16S |
| 16S R | CCCGAAGGCCGTCATCCCTCACGC | Reverse RT-PCR amplification of RNA16S |
