# Supplementary figures and images for: Novel Two-Component Systems Implied in Antibiotic Production in Streptomyces coelicolor
Source: PLoS One. 2011 May 20;6(5):e19980. doi: 10.1371/journal.pone.0019980 (PMC3098853; doi:10.1371/journal.pone.0019980)

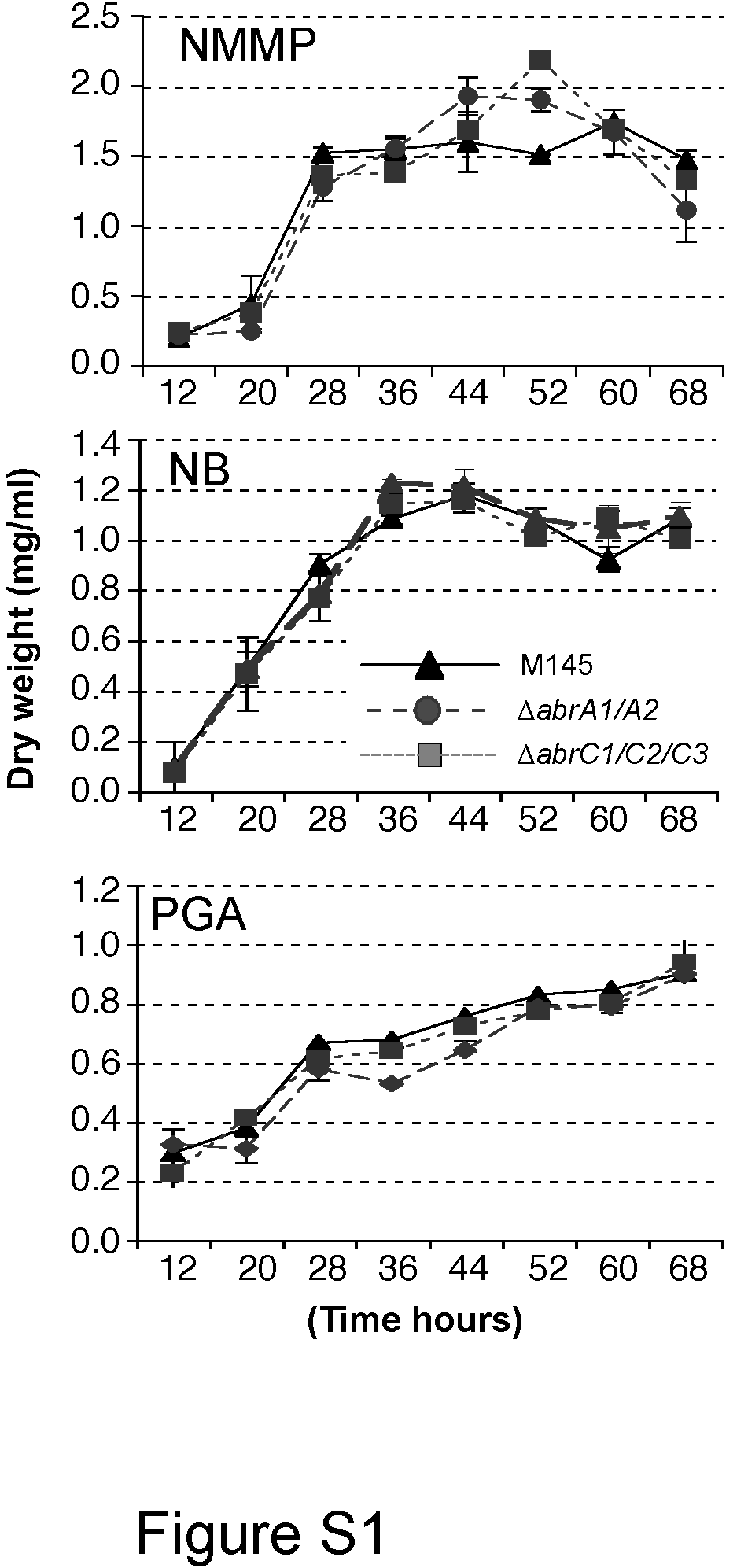

Supplement: Figure S1 — Growth curves of the different strains in NMMP (A), NB (B) and PGA (C) S. coelicolor M145 (triangles), S. coelicolor ΔabrA1/A2 (circles) and S. coelicolor ΔabrC1/C2/C3 (squares). Error bars correspond to standard deviation of two independent experiments measured by duplicate. (TIF) [file pone.0019980.s004.tif]

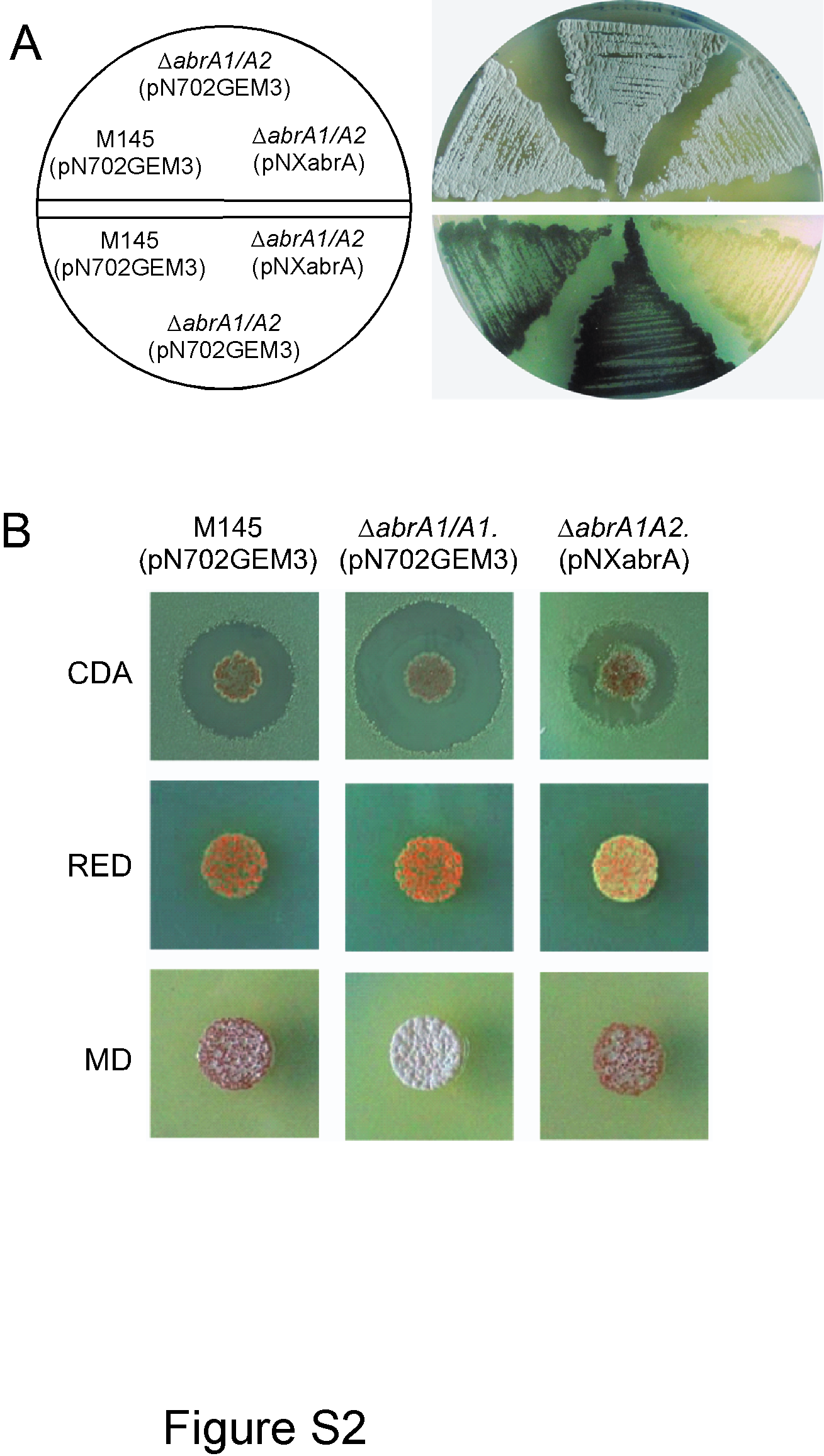

Supplement: Figure S2 — Phenotypes of strains expressing abrA1/A2 in multicopy plasmid. A: Effect of the expression of abrA1/A2 genes by the high copy number plasmid pNXabrA derived from pN702GEM3 on NMMP medium. Top: morphological differentiation. Bottom: ACT production. B: CDA bioassays on NA medium, RED production on PGA medium, and morphological differentiation on YEPD medium (2 days), in the different strains. (TIF) [file pone.0019980.s005.tif]

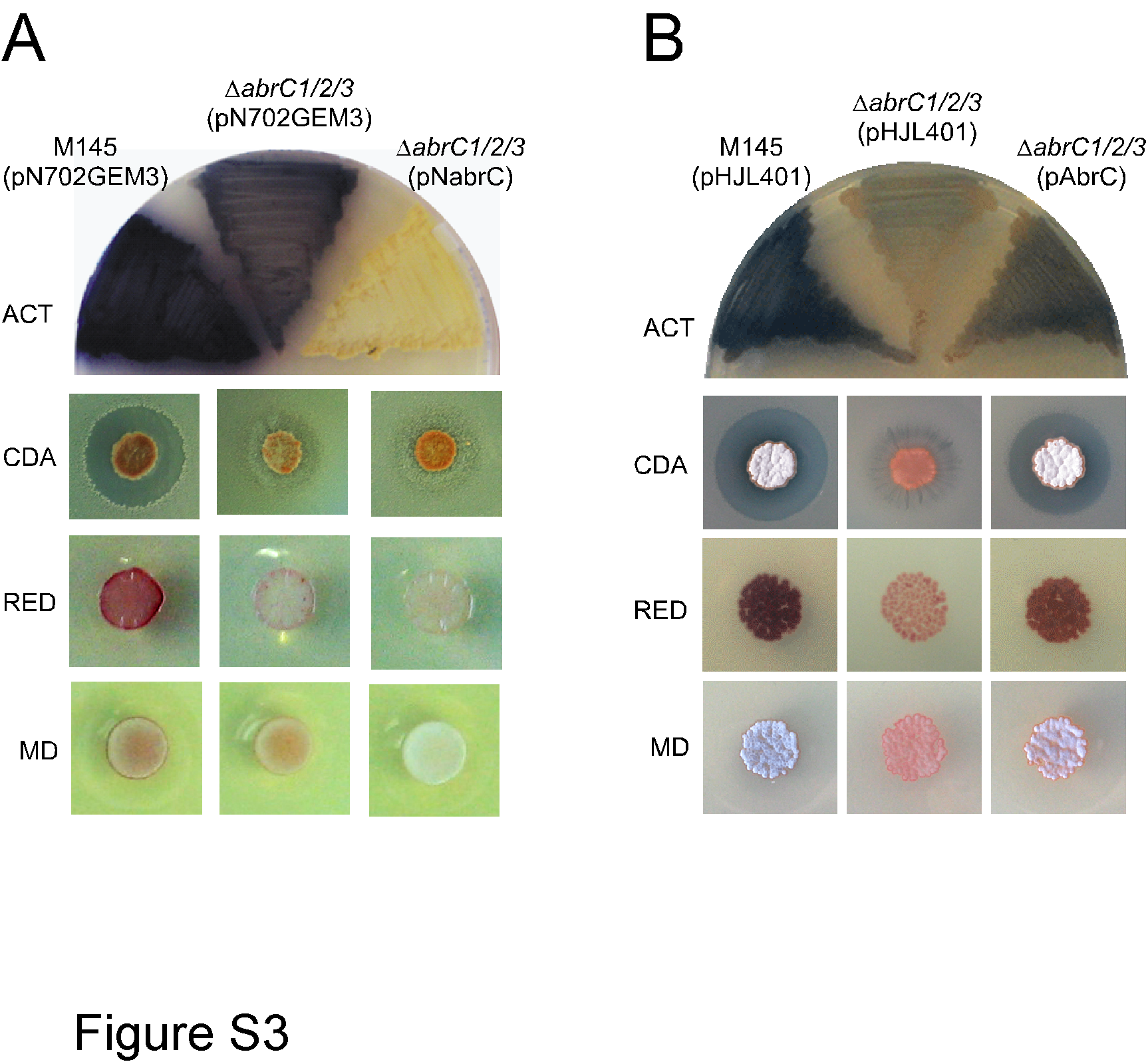

Supplement: Figure S3 — Phenotypes of strains expressing abrC1/C2/C3 in multicopy plasmids. A: Effect of expression of abrC1/C2/C3 genes by the high copy number plasmid pNabrC derived from pN702GEM3: ACT production on NA medium, CDA bioassays on NA medium, RED production on PGA medium, and MD morphological differentiation on YEPD medium (2 days), by the different strains. B: Effect of expression of abrC1/C2/C3 genes by the low copy number pAbrC plasmid derived from pHJL401: ACT production on NA medium, CDA bioassays on NA medium, RED production on PGA medium, and MD morphological differentiation on YEPD medium (3 days), by the different strains. (TIF) [file pone.0019980.s006.tif]
